# Comment on Yang et al. Bile Acid Diarrhea: From Molecular Mechanisms to Clinical Diagnosis and Treatment in the Era of Precision Medicine. *Int. J. Mol. Sci.* 2024, *25*, 1544

**DOI:** 10.3390/ijms25158047

**Published:** 2024-07-24

**Authors:** Anne-Marie Ellegaard, Martin L. Kårhus, Filip K. Knop

**Affiliations:** 1 Center for Clinical Metabolic Research, Copenhagen University Hospital—Herlev and Gentofte, DK-2900 Hellerup, Denmark; 2 Clinical Research, Steno Diabetes Center Copenhagen, DK-2730 Herlev, Denmark; 3 Department of Clinical Medicine, The Faculty of Health and Medical Sciences, Copenhagen University, DK-2200 Copenhagen, Denmark

We have with great interest read the recent review on the molecular mechanisms underlying bile acid diarrhea (BAD) by Yang et al. [1]. The authors review the functional implications of several key components of the bile acid metabolism, such as the farnesoid X receptor, the Takeda G-protein receptor 5, and fibroblast growth factor 19, in BAD. Furthermore, the current and potential diagnostic methods are briefly summarized.

In the last section, the existing treatment options for BAD are reviewed, including bile acid sequestrants and farnesoid X receptor agonists. We agree that these therapies are important in BAD treatment. However, we find that the authors have overlooked the evidence and clinical use of liraglutide in BAD management. In a randomized clinical trial with 52 individuals with primary or post-cholecystectomy BAD treated for six weeks with either colesevelam (1875 mg twice daily) or liraglutide (once-daily subcutaneous injection uptitrated from 0.6 mg to 1.8 mg daily over three weeks), we found that both treatments effectively and safely reduced the number of daily stools and the number of watery stools [2]. Importantly, we showed that the liraglutide treatment was superior to colesevelam in reducing the BAD symptoms, assessed as a reduction in daily stools of ≥25% [2]. Despite the relatively small sample size and the current lack of confirmatory studies, this study provides, to our knowledge, the most solid clinical evidence for any BAD treatment. Following the publication of this study in 2022, an overwhelming number of primary care physicians, gastroenterologists, as well as patients have started using—or expressed a desire to start using—liraglutide in BAD management. Recently, we published a demographic characterization of BAD in Denmark including register data retrieved up to and including 2021 [3]. In this study, we showed that 248 individuals used liraglutide after a diagnostic SeHCAT test [3]. Notably, these individuals did not use liraglutide prior to the test, suggesting that liraglutide was prescribed for BAD treatment [3].

Overall, despite the sparse literature on liraglutide treatment for BAD (apart from the studies mentioned here, there are a few case reports [4,5,6] and a register-based analysis [7]), we find it peculiar that the authors of the review have omitted this treatment from their review of the BAD treatment options since this treatment has been tested in the longest clinical trial regarding BAD treatment and the clinical use of liraglutide to manage BAD is increasing. In our opinion, it is important to share all the knowledge on the best treatment options with both scientists and clinicians to inspire scientific progress and improve the clinical management to the benefit of the patients.
